# Epigenetic Alteration Shaped by the Environmental Chemical Bisphenol A

**DOI:** 10.3389/fgene.2020.618966

**Published:** 2021-01-11

**Authors:** Tengfei Qin, Xiaoping Zhang, Ting Guo, Ting Yang, Yahui Gao, Wei Hao, XiangFen Xiao

**Affiliations:** ^1^Henan Collaborative Innovation Center of Modern Biological Breeding, Henan Institute of Sciences and Technology, Xinxiang, China; ^2^School of Medical Science, Chifeng University, Chifeng, China; ^3^Department of Nephrology, Affiliated Hospital of Beihua University, Jilin City, China; ^4^School of Life Sciences, The Chinese University of Hong Kong, Shatin, China; ^5^Shanghai Institutes for Biological Sciences, Chinese Academy of Sciences, Shanghai, China

**Keywords:** bisphenol A, epigenetic alteration, DNA methylation, histone modifications, toxico-epigenomics

## Abstract

Bisphenol A (BPA) is extensively used in plastic products and epoxy resins. The epigenetic response to the environmental chemical BPA was involved in multiple dysfunctional categories, such as cancer, the reproductive system, metabolism, pubertal development, peripheral arterial disease, infant and childhood growth, and neurodevelopment outcomes. In this mini-review, we described the recent progress of the epigenetic effects of the environmental chemical BPA, including DNA methylation, histone methylation, and toxic epigenomics. Notably, the histone modification changes under BPA exposure are summarized in this review. DNA methylation accompanied by transcriptional changes in key genes affected by BPA exposure is related to various processes, including neural development, cancer pathways, and generational transmission. In addition, BPA could also affect histone modifications in many species, such as humans, rats, and zebrafish. Finally, we reviewed recent studies of the toxico-epigenomics approach to reveal the epigenetic effect of BPA exposure genome-wide.

## Introduction

Bisphenol A (BPA) is one of the most extensively used chemical components in the plastics and epoxy resins of large consumer products, including water bottles, baby bottles and storage containers, pitchers, tableware, and other storage goods (Kamrin, 2004). Almost everyone can be exposed to BPA every day from the environment, especially from food intake. BPA has been detected in multiple biological samples, including urine, blood, saliva, and even breast milk (Vandenberg et al., 2010).

Accumulating evidence shows that BPA, an endocrine-disrupting chemical (EDC), interferes with hormonal signaling pathways, which is acutely toxic to living organisms (Diamanti-Kandarakis et al., 2009). In recent years, many adverse effects on the reproductive system have been found to be associated with BPA exposure in both animals and humans (Shankar et al., 2012; Adeyi and Babalola, 2019).

Epidemiological studies discovered that cancer, reproductive system, metabolism, pubertal development, peripheral arterial disease, infant and childhood growth, and neurodevelopment outcomes were associated with BPA exposure (Matuszczak et al., 2019). For example, urinary BPA concentrations were reported to be associated with serum reproductive hormones. Compared with unexposed BPA workers, exposed workers were more likely to suffer from male sexual dysfunction (Braun and Hauser, 2011). Urinary BPA levels were significantly correlated with peripheral arterial disease based on the analysis of representative samples (Shankar et al., 2012).

Bisphenol A exposure can induce epigenetic misregulation, especially early persistent exposure, which can affect the development of offspring. Epigenetic changes, including modifications in DNA, RNA, and histones combined with noncoding RNA (ncRNA) and miRNA-associated gene silencing, are currently thought to initiate and sustain transcriptional regulation without alteration of DNA sequencing (Weinhold, 2006). Compared to genetic alterations, epigenetic regulation is more dynamic in response to environmental changes and environmental exposures. The major DNA modification is DNA methylation of 5-mC in cytosine in both symmetric and asymmetric contexts (CG, CHG, and CHH). Oxidation of methylated cytosine (5-mC), methylation of adenine (A), and other modifications are also regarded as significant epigenetic regulators (Kumar et al., 2018). Histone modification is a covalent posttranslational modification (PTM) of histones. Histone modification of acetylation and methylation on histone H3 could regulate chromatin structure and transcription (Bannister and Kouzarides, 2011). Epigenetic modifications are tightly controlled during human development, especially in the very early stages from fertilization to birth. ncRNAs are a group of functional RNA molecules transcribed from DNA but not translated into proteins (Wei et al., 2017). ncRNAs have been found to be involved in various processes, such as histone modification, DNA methylation targeting, and gene silencing. Numerous miRNAs have been shown to play significant roles in virtually every cellular process and are required for animal development, cell differentiation, and homeostasis.

To conduct our review on the most recent studies about the epigenetic effects of BPA, we used a combination of the key words “bisphenol A” AND “epigenetic” OR “DNA methylation” OR “histone OR “miRNA” to search the PubMed database from 2012 to August 2020. In total, we found approximately 200 papers and manually checked each article. We then classified them into three categories: DNA methylation, histone modification, and both DNA methylation and histone methylation. In addition, we further selected studies with high-throughput sequencing data, such as toxico-epigenomics study cases. Because a comprehensive review has only published the miRNA changes induced by BPA exposure, we will not review the miRNA and ncRNA parts in this review (Sabry et al., 2019; Farahani et al., 2020).

New and ongoing studies are continuously revealing the role of epigenetics in human disorders and fatal diseases linked to environmental exposure, including endocrine disruptors (BPA, DDT, phthalates; Dutta et al., 2020); tobacco smoke (Wu et al., 2019); diesel exhaust particles, such as PM2.5 and PM10; heavy metals, such as arsenic, nickel, and lead (Wang et al., 2020); and other indoor and outdoor pollutants (Mileva et al., 2014; Zhu et al., 2018; Freeman et al., 2020). A systematic review summarized that BPA has many effects, such as hormonal alterations, alteration of male reproductive organs, and epigenetic changes involving the male reproductive system (Cariati et al., 2020). In this review, we summarized the recent discoveries of BPA-mediated epigenetic alterations in DNA methylation and histone modification. In addition, we also reviewed toxico-epigenomics studies on different kinds of animal models.

## BPA and DNA Methylation

Bisphenol A could affect DNA methylation as an endocrine-disrupting chemical. In Table 1, we summarized the genes that have been reported to be methylated or demethylated under BPA treatment since 2012, while another review reported BPA-affected genes epigenetically until 2012 (Singh and Li, 2012).

**Table 1:** DNA hypomethylation and hypermethylation induced by bisphenol A (BPA).

Genes	Epigenetic alterations	Treatment methods	Target tissue	References
*ERα*	DNA hypomethylation	BPA given to female mice during pregnancy	Brain of F1 offspring mice	Kundakovic et al. (2013)
*BDNF*	DNA hypomethylation	Pregnant mice were orally treated with BPA	Blood of female mice and hippocampus of F1 mice	Kundakovic et al. (2015)
*Igf2*	DNA hypermethylation	Pregnant F0 SD rats were orally treated with BPA	Sperm of adult F1 male rats and male F2 pancreatic beta-cells	Mao et al. (2015)
	DNA hypermethylation	Early-life exposure to BPA and/or variable diet	paired mouse tail tissue	Kochmanski et al. (2017)
*Stat3*	DNA hypermethylation	Liver tissue exposed to BPA	Fetal liver of mice and humans	Weinhouse et al. (2015)
*MEST*	DNA hypermethylation	Prenatal BPA potential exposure	Human cord blood and visceral fat tissue of the offspring of BPA-exposed dams	Junge et al. (2018)
*Grin2b*	DNA hypomethylation	Prenatal BPA potential exposure	Hippocampus of female rats and humans	Alavian-Ghavanini et al. (2018)
*TET2*	DNA hydroxymethylation	MCF-7 cells treated with BPA or bisphenol S (BPS)	Breast cancer cells	Li et al. (2020)
*Scgb2a1*	DNA hypomethylation	BPA oral route of exposure	Adult rat prostate	Wong et al. (2015)

Bisphenol A was extensively found to be involved in neurodevelopment by affecting DNA methylation. Kundakovic et al. (2013) found that *in utero* BPA exposure induced sex-specific DNA methylation changes in *ERα* [estrogen receptor 1 (*Esr1*)] in the brain. Furthermore, they detected enduring DNA methylation changes in brain-derived neurotrophic factor (*BDNF*), a specific gene related to nervous system development, that were induced by parental BPA exposure (Kundakovic et al., 2015). These results indicated that BPA exposure-induced DNA methylation changes in specific genes may underlie long-lasting effects on brain function, behavior, and neurodevelopment. Another group of scientists found that, by binding to tyrosine kinase B (TrkB), BDNF induces TrkB autophosphorylation, which activates the RAS-MAPK pathway, and finally, CREB (cAMP responsive element binding protein) is activated at the serine site. By increasing the expression of *BDNF* and the antiapoptotic gene *Bcl-2*, CREB could increase the survival of nerve cells, synaptic plasticity, and neurogenesis. A BPA or ethinyl estradiol (EE) consortium study revealed that exposure to BPA or EE may lead to *BDNF* gene expression. Exposure may also lead to changes in DNA methylation in the hippocampus and hypothalamus of adult rats. The changes in *BDNF* in the hippocampus exhibited some connections with the performance of female mice in the Barnes maze (Cheong et al., 2018). The *Grin2b* gene is important for the regulation of neural morphology, learning, and memory. Genetic polymorphisms in Grin2b have been demonstrated to be involved in neurodevelopmental diseases and disorders. Alavian-Ghavanini et al. (2018) found that prenatal BPA exposure is connected with epigenetic changes in *Grin2b* in female rats and humans. In addition, the global levels of 5-mC and 5-hmC were significantly increased after BPA treatment in human neuroblastoma cells (SH-SY5Y; Senyildiz et al., 2017).

Cancer cell proliferation has been reported to be mediated by DNA methylation induced by BPA exposure. Recently, Li et al. (2020) revealed the pivotal role of TET dioxygenases and DNA hydroxymethylation in the bisphenol-stimulated proliferation of breast cancer cells. They found that BPA or its replacement BPS increased the promoter methylation of TET2, leading to an inhibition of TET2 expression and DNA hydroxymethylation. A decrease in DNA hydroxymethylation induces increased proliferation of ER-positive breast cancer cells. Secretoglobins are a family of small secreted proteins and are thought to be involved in many processes, such as inflammation, tissue repair, and tumorigenesis. Wong et al. (2015) used a rat model for the developmental reprogramming of susceptibility to prostate carcinogenesis. Using RNA-seq, they found that the gene expression of *Scgb2a1* was significantly upregulated in the prostate of adult rats neonatally exposed to BPA. DNA methylation analysis of the *Scgb2a1* promoter revealed significant CpG island hypomethylation upstream of the TSS of *Scgb2a1* in the reprogrammed prostate. Their results indicate that BPA exposure could reprogram *Scgb2a1* expression in the adult prostate epigenetically during the development of the prostate (Wong et al., 2015).

In another study, the DREAM (digital restriction enzyme analysis of methylation) method was also applied in the zebrafish embryo for the study of DNA methylation modifications. Site-specific methylation in the promoter of the vasa gene was more responsive in the three examined genes, indicating that the vasa gene could be a potential marker for BPA exposure in zebrafish (Bouwmeester et al., 2016). Signal transducer and activator of transcription 3 (Stat3) is an essential transcription factor and plays roles in immune signal translocation. From the mouse fetal liver, DNA methylation profiles within STAT3 changed following the BPA level in human fetal liver samples, indicating that STAT3 could serve as a translationally relevant candidate biomarker (Weinhouse et al., 2015).

Transposable elements accounted for 45 and 37.5% of the human genome and mouse genome, respectively. Upon BPA exposure, Faulk et al. (2016) found remarkable DNA methylation changes in transposons in the human liver (1,251 individual transposon loci), while they did not detect significantly detectable differential DNA methylation in mice (only 19 loci were identified).

Recent studies also showed that BPA exposure epigenetically affected imprinted genes. Insulin-like growth factor-2 (Igf2) is a critical imprinted gene (Morison and Reeve, 1998) that is mainly regulated by differentially methylated regions (DMRs; Portela and Esteller, 2010). Kochmanski et al. (2017) found that BPA exposure in the diet showed no significant effect on the methylation drift at *LINE-1*, *IAP*, *H19*, or *Esr1* but presented a marginally significant effect on age-related methylation at *Igf2*. Mao et al. (2015) revealed that the hypermethylation of Igf2 in islets was involved in generational transmission of glucose intolerance and pancreatic beta-cell impairment in F2 offspring induced by F1 early life BPA exposure. These DNA methylation changes in germ cells may facilitate generational transmission. However, in one study using mouse models, van Esterik et al. (2015) concluded that although different metabolic phenotypes in female offspring after maternal BPA exposure were observed, changes in DNA methylation could not explain the abnormal metabolic phenotypes. BPA can disrupt human placental epigenetic modifications, including genomic imprinting, DNA methylation, and the expression of ncRNAs (Strakovsky and Schantz, 2018). Furthermore, Kochmanski et al. (2018) profiled genome-wide 5-hmC levels and discovered that perinatal BPA exposure induced persistent 5-hmC markers at multiple imprinted loci in mouse blood during development.

These recent discoveries indicate that BPA exposure could affect DNA methylation and hydroxymethylation at multiple loci related to the neural system, cancer cell proliferation, and imprinted genes and could further affect the expression levels and their functions in the cell.

## BPA and Histone Modifications

Apart from DNA methylation, histone modifications are another type of epigenetic marker for transcriptional regulation. Sometimes changes in histone modifications may accompany DNA methylation. Here, we reviewed the recent findings of histone modification changes under BPA treatment.

For example, BPA could alter the histone H3K4me3, histone acetylation, and RNA polymerase II (RNAP II) recruitment to the long noncoding RNA (lncRNA) HOTAIR in human breast cancer cells (MCF7) and in the mammary glands of rats (Bhan et al., 2014a). As another example, enhancer of Zeste homolog 2 (EZH2), a histone methyltransferase specific to H3K27, was found to be transcriptionally induced by the estrogenic endocrine disruptor BPA in MCF7 cells and the mammary glands of ovariectomized (OVX) rats (Bhan et al., 2014b).

Furthermore, HOXC6 and HOXB9 are homeobox-containing genes related to mammary gland development that are overexpressed in various cancers. BPA treatment induced the expression of HOXC6 and HOXB9 in human breast cancer cells (MCF7) and the mammary glands of ovariectomized (OVX) rats. Luciferase assays indicated that estrogen-response elements (EREs) located in the HOXB9/HOXC6 promoter are irreplaceable for BPA-induced expression. Estrogen receptors (ERs) and other ER coregulators can bind to the HOXB9/HOXC6 promoter in the presence of BPA, resulting in increased chromatin H3K4me3, histone acetylation, and gene activation. It is possible that the increase in H3K4me3, histone acetylation, and recruitment of RNAP II at the HOXC6/HOXB9 promoters was caused by exposure to BPA (Hussain et al., 2015; Deb et al., 2016). In another study, BPA induced a significant decrease in H3K9ac and H3K9me3 levels overall in SH-SY5Y cells (Senyildiz et al., 2017).

BPA exposure could affect porcine oocyte maturation. Wang et al. (2016) found that the oocyte maturation rate was significantly reduced with 250 μM BPA treatment *in vitro*. The intensity of H3K4me2 and the 5-mC levels were decreased after BPA treatment, suggesting that disturbed epigenetic modification might inhibit the meiotic progression of oocytes. Using *Chironomus riparius* as an animal model, Lee et al. (2018) observed some potential interactions between altered H3K36 and metabolites caused by BPA exposure.

Exposure to BPA could impair primordial germ cell migration. *cxcr4b* and *sdf1a* are two key genes involved in PGC migration. Lombo et al. (2019) found that cxcr4b and sdf1a were highly dysregulated in zebrafish embryos exposed to BPA. Within the genital ridge, no significant changes were determined in germ or somatic cells. However, the males exhibited a reduced H3K9ac level in sperm with embryonic BPA exposure. In another study using male zebrafish, a high dose of BPA affected spermatocyte function. The mRNA level of epigenetic remodeling enzymes in testes was misregulated. BPA also triggered the activity of histone acetyltransferase (HAT), resulting in increased levels of H3K9ac, H3K14ac, and H4K12ac (Gonzalez-Rojo et al., 2019).

In summary, BPA exposure could affect histone modification in humans, rats, porcines, *C. riparius*, and zebrafish accompanied by impairment of primordial germ cell migration.

## BPA and the Toxico-Epigenomics

Toxicogenomics utilizes comprehensive gene expression data to profile gene expression features that strongly correlate with genetic toxicity (Suter et al., 2004; Gomase and Tagore, 2008). Indeed, with the development of high-throughput sequencing technology, apart from gene expression, DNA methylation, histone modification, or even RNA modification could be detected at the genome-wide scale to reveal the epigenetic response to toxins and environmental pollutants. Toxico-epigenomics emerged from the combination of epigenomics technology and classical toxicology. Using the toxico-epigenomics approach, we can study epigenetic alterations at specific loci under environmental exposure and the role of the epigenome as a possible mediator of the exposure effect (Chung and Herceg, 2020).

Faulk et al. (2015) compared the DNA methylation levels of three groups with different BPA concentrations. They found that BPA levels were positively and negatively associated with methylation in CpG islands and CpG shores, respectively. They also detected hypermethylated regions at the SNORD115 loci.

Using a mouse model, Weinhouse et al. (2014) revealed the dose-dependent incidence of hepatic tumors in adult mice following perinatal exposure to BPA. While the mechanism of the effect is unclear, they performed epigenome-wide DNA methylation profiles using DNA methylation arrays of adult mouse livers to reveal the methylation changes associated with perinatal BPA exposure and disease in mice with and without liver tumors. They proposed that the Jak/Stat and Mapk signaling pathways would be altered in comparisons of cancer and normal tissues. On the other hand, these Jak/Stat and Mapk pathway-related genes were differentially methylated between different doses of BPA (Weinhouse et al., 2016).

Multiple studies have found that BPA exposure could change the epigenetics of the offspring of exposed parents using low-throughput methods. Aiba et al. (2018) found that there was no significant difference in methylation levels in any CpG site in the control and low-dose BPA-treated groups. This suggested that the effect of low-dose BPA exposure on hippocampal DNA methylation levels at the fetal stage is extremely small.

Jadhav et al. (2017) employed genome-wide methyl-binding domain sequencing of the breasts of 100-day-old rats exposed prepubertally to BPA. They found that altered DNA methylation under BPA exposure can effectively provide predictive value for poor survival in TCGA breast cancer patients.

Bisphenol A has been thought to be related to obesity and diabetes. Using a mouse model, Anderson et al. (2013) revealed that in adult female mice, maternal dietary BPA exposure altered metabolic phenotypes, such as lowering body fat and hormone homeostasis. To elucidate the epigenetic mechanisms related to the metabolic changes, they applied the MBD-enriched DNA fragment and hybridized it to the DNA methylation array. Through bioinformatic analysis, they found that the differentially methylated genes were enriched in metabolic pathways. Furthermore, four candidate genes (*Jak-2*, *Rxr*, *Rfxap*, and *Tmem238*) were selected to assess DNA methylation as a mediating factor that connects perinatal BPA exposure to metabolic phenotypes. DNA methylation status at the four genes was used in mediational regression analysis and was recognized as a mediator in the mechanistic pathway of developmental BPA exposure (Anderson et al., 2017).

BPA exposure has been reported to change behavior in fish models. One study showed that alternating light stimulation with BPA could obviously decrease the zebrafish swimming speed. Olsvik et al. (2019) applied WGBS analysis to fish exposed to BPA and found that *dnmt1* and *cbs* may be affected by BPA. They did not detect a significant effect on global DNA methylation but found that, in 4,873 genes, there were 20,474 differentially methylated (DM) sites. Some of the DM sites occurred in two protocadherin *γ*-subfamily genes related to axon targeting. Their data revealed that low-dose BPA exposure could affect the methylation patterns of genes associated with the nervous system and finally affect swimming speed.

In summary, most of the time, BPA exposure could be involved in changes in DNA methylation at the whole genome level in rat, mouse, and fish models. The changes in DNA methylation locus sites were enriched in cancer, metabolism, and nervous system pathways.

## Conclusions and Perspectives

As one of the most widely used chemical components, BPA was reported to influence the dysfunction of different aspects. Although multiple animal studies and epidemiological studies have shown that BPA could be involved in various diseases, the mechanisms of how BPA affects the disease are still not clear. Epigenetic changes, including DNA methylation, histone modification, and ncRNA, have been demonstrated to be related to disease development under environmental exposure. We reviewed the recent progress of epigenetic changes under BPA exposure. BPA exposure could affect the DNA methylation of multiple genes that could contribute to brain development, cancer progression, and important signaling pathways. On the other hand, histone modifications also change significantly at some loci upon BPA exposure.

High-throughput sequencing data from epigenetic profiling of toxic exposure enables us to study the epigenetic mechanism of the toxicological effect of environmental exposure. Recently, epigenomics approaches, including WGBS, ChIP-seq, and ATAC-seq, have been employed to explore the epigenetic regulation of environmental exposure. However, the majority of the studies were focused on DNA methylation changes under BPA exposure. Recently, more systematic data from the exposure were published for the community to explore. For example, the National Institute of Environmental Health Sciences (NIEHS) launched Toxicant Exposures and Responses by Genomic and Epigenomic Regulators of Transcription (TaRGET) to study the transcriptional and epigenetic regulation of environmental toxins, including arsenite, BPA, DEHP, Pb, and TBT (Wang et al., 2018). With the development of new technologies, the cost of high-throughput sequencing will decrease significantly. We will see an increasing number of studies on the effects of BPA, which will help us to better understand the epigenetic changes related to diseases induced by BPA. Additionally, further investigations in this field will facilitate our understanding of the pathogenesis of these diseases, thus helping in the treatment of diseases associated with BPA exposure.

## Author Contributions

XX and WH contributed equally to the design and coordination of the study. TQ, XZ, and XX collected the data; with the help from the TG and TY, YG, XX, WH, TQ, and XZ wrote the manuscript. All authors contributed to the article and approved the submitted version.

### Conflict of Interest

The authors declare that the research was conducted in the absence of any commercial or financial relationships that could be construed as a potential conflict of interest.

## References

[ref1] Adeyi A. A. Babalola B. A. (2019). Bisphenol-A (BPA) in foods commonly consumed in Southwest Nigeria and its human health risk. Sci. Rep. 9:17458. 10.1038/s41598-019-53790-2, PMID: 31767906 PMC6877615

[ref2] Aiba T. Saito T. Hayashi A. Sato S. Yunokawa H. Maruyama T.. (2018). Does the prenatal bisphenol A exposure alter DNA methylation levels in the mouse hippocampus?: an analysis using a high-sensitivity methylome technique. Genes Environ. 40:12. 10.1186/s41021-018-0099-y, PMID: 29881475 PMC5985587

[ref3] Alavian-Ghavanini A. Lin P. I. Lind P. M. Risen Rimfors S. Halin Lejonklou M. Dunder L.. (2018). Prenatal bisphenol A exposure is linked to epigenetic changes in glutamate receptor subunit gene Grin2b in female rats and humans. Sci. Rep. 8:11315. 10.1038/s41598-018-29732-9, PMID: 30054528 PMC6063959

[ref4] Anderson O. S. Kim J. H. Peterson K. E. Sanchez B. N. Sant K. E. Sartor M. A.. (2017). Novel epigenetic biomarkers mediating bisphenol A exposure and metabolic phenotypes in female mice. Endocrinology 158, 31–40. 10.1210/en.2016-1441, PMID: 27824486 PMC5412976

[ref5] Anderson O. S. Peterson K. E. Sanchez B. N. Zhang Z. Mancuso P. Dolinoy D. C. (2013). Perinatal bisphenol A exposure promotes hyperactivity, lean body composition, and hormonal responses across the murine life course. FASEB J. 27, 1784–1792. 10.1096/fj.12-223545, PMID: 23345456 PMC3606526

[ref6] Bannister A. J. Kouzarides T. (2011). Regulation of chromatin by histone modifications. Cell Res. 21, 381–395. 10.1038/cr.2011.22, PMID: 21321607 PMC3193420

[ref7] Bhan A. Hussain I. Ansari K. I. Bobzean S. A. Perrotti L. I. Mandal S. S. (2014a). Bisphenol-A and diethylstilbestrol exposure induces the expression of breast cancer associated long noncoding RNA HOTAIR in vitro and in vivo. J. Steroid Biochem. Mol. Biol. 141, 160–170. 10.1016/j.jsbmb.2014.02.002, PMID: 24533973 PMC4025971

[ref8] Bhan A. Hussain I. Ansari K. I. Bobzean S. A. Perrotti L. I. Mandal S. S. (2014b). Histone methyltransferase EZH2 is transcriptionally induced by estradiol as well as estrogenic endocrine disruptors bisphenol-A and diethylstilbestrol. J. Mol. Biol. 426, 3426–3441. 10.1016/j.jmb.2014.07.025, PMID: 25088689

[ref9] Bouwmeester M. C. Ruiter S. Lommelaars T. Sippel J. Hodemaekers H. M. van den Brandhof E. J.. (2016). Zebrafish embryos as a screen for DNA methylation modifications after compound exposure. Toxicol. Appl. Pharmacol. 291, 84–96. 10.1016/j.taap.2015.12.012, PMID: 26712470

[ref10] Braun J. M. Hauser R. (2011). Bisphenol A and children’s health. Curr. Opin. Pediatr. 23, 233–239. 10.1097/MOP.0b013e3283445675, PMID: 21293273 PMC6028937

[ref11] Cariati F. Carbone L. Conforti A. Bagnulo F. Peluso S. R. Carotenuto C.. (2020). Bisphenol A-induced epigenetic changes and its effects on the male reproductive system. Front. Endocrinol. 11:453. 10.3389/fendo.2020.00453, PMID: 32849263 PMC7406566

[ref12] Cheong A. Johnson S. A. Howald E. C. Ellersieck M. R. Camacho L. Lewis S. M.. (2018). Gene expression and DNA methylation changes in the hypothalamus and hippocampus of adult rats developmentally exposed to bisphenol A or ethinyl estradiol: a CLARITY-BPA consortium study. Epigenetics 13, 704–720. 10.1080/15592294.2018.1497388, PMID: 30001178 PMC6224219

[ref13] Chung F. F. Herceg Z. (2020). The promises and challenges of toxico-epigenomics: environmental chemicals and their impacts on the epigenome. Environ. Health Perspect. 128:15001. 10.1289/EHP6104, PMID: 31950866 PMC7015548

[ref14] Deb P. Bhan A. Hussain I. Ansari K. I. Bobzean S. A. Pandita T. K.. (2016). Endocrine disrupting chemical, bisphenol-A, induces breast cancer associated gene HOXB9 expression in vitro and in vivo. Gene 590, 234–243. 10.1016/j.gene.2016.05.009, PMID: 27182052 PMC4975976

[ref15] Diamanti-Kandarakis E. Bourguignon J. P. Giudice L. C. Hauser R. Prins G. S. Soto A. M.. (2009). Endocrine-disrupting chemicals: an endocrine society scientific statement. Endocr. Rev. 30, 293–342. 10.1210/er.2009-0002, PMID: 19502515 PMC2726844

[ref16] Dutta S. Haggerty D. K. Rappolee D. A. Ruden D. M. (2020). Phthalate exposure and long-term epigenomic consequences: a review. Front. Genet. 11:405. 10.3389/fgene.2020.00405, PMID: 32435260 PMC7218126

[ref17] Farahani M. Rezaei-Tavirani M. Arjmand B. (2020). A systematic review of microRNA expression studies with exposure to bisphenol A. J. Appl. Toxicol. 41, 4–19. 10.1002/jat.4025, PMID: 32662106

[ref18] Faulk C. Kim J. H. Anderson O. S. Nahar M. S. Jones T. R. Sartor M. A.. (2016). Detection of differential DNA methylation in repetitive DNA of mice and humans perinatally exposed to bisphenol A. Epigenetics 11, 489–500. 10.1080/15592294.2016.1183856, PMID: 27267941 PMC4939917

[ref19] Faulk C. Kim J. H. Jones T. R. McEachin R. C. Nahar M. S. Dolinoy D. C.. (2015). Bisphenol A-associated alterations in genome-wide DNA methylation and gene expression patterns reveal sequence-dependent and non-monotonic effects in human fetal liver. Environ. Epigenet. 1:dvv006. 10.1093/eep/dvv006, PMID: 27358748 PMC4922640

[ref20] Freeman D. M. Lou D. Li Y. Martos S. N. Wang Z. (2020). The conserved DNMT1-dependent methylation regions in human cells are vulnerable to neurotoxicant rotenone exposure. Epigenet. Chromatin 13:17. 10.1186/s13072-020-00338-8, PMID: 32178731 PMC7076959

[ref21] Gomase V. S. Tagore S. (2008). Toxicogenomics. Curr. Drug Metab. 9, 250–254. 10.2174/138920008783884696, PMID: 18336230

[ref22] Gonzalez-Rojo S. Lombo M. Fernandez-Diez C. Herraez M. P. (2019). Male exposure to bisphenol A impairs spermatogenesis and triggers histone hyperacetylation in zebrafish testes. Environ. Pollut. 248, 368–379. 10.1016/j.envpol.2019.01.127, PMID: 30818116

[ref23] Hussain I. Bhan A. Ansari K. I. Deb P. Bobzean S. A. Perrotti L. I.. (2015). Bisphenol-A induces expression of HOXC6, an estrogen-regulated homeobox-containing gene associated with breast cancer. Biochim. Biophys. Acta 1849, 697–708. 10.1016/j.bbagrm.2015.02.003, PMID: 25725483 PMC4437882

[ref24] Jadhav R. R. Santucci-Pereira J. Wang Y. V. Liu J. Nguyen T. D. Wang J.. (2017). DNA methylation targets influenced by bisphenol A and/or genistein are associated with survival outcomes in breast cancer patients. Gen. Dent. 8:144. 10.3390/genes8050144, PMID: 28505145 PMC5448018

[ref25] Junge K. M. Leppert B. Jahreis S. Wissenbach D. K. Feltens R. Grutzmann K.. (2018). MEST mediates the impact of prenatal bisphenol A exposure on long-term body weight development. Clin. Epigenetics 10:58. 10.1186/s13148-018-0478-z, PMID: 29721103 PMC5910578

[ref26] Kamrin M. A. (2004). Bisphenol A: a scientific evaluation. MedGenMed 6:7., PMID: 15520629 PMC1435609

[ref27] Kochmanski J. Marchlewicz E. H. Savidge M. Montrose L. Faulk C. Dolinoy D. C. (2017). Longitudinal effects of developmental bisphenol A and variable diet exposures on epigenetic drift in mice. Reprod. Toxicol. 68, 154–163. 10.1016/j.reprotox.2016.07.021, PMID: 27496716 PMC5290281

[ref28] Kochmanski J. J. Marchlewicz E. H. Cavalcante R. G. Perera B. P. U. Sartor M. A. Dolinoy D. C. (2018). Longitudinal effects of developmental bisphenol A exposure on epigenome-wide DNA hydroxymethylation at imprinted loci in mouse blood. Environ. Health Perspect. 126:077006. 10.1289/EHP3441, PMID: 30044229 PMC6108846

[ref29] Kumar S. Chinnusamy V. Mohapatra T. (2018). Epigenetics of modified DNA bases: 5-methylcytosine and beyond. Front. Genet. 9:640. 10.3389/fgene.2018.00640, PMID: 30619465 PMC6305559

[ref30] Kundakovic M. Gudsnuk K. Franks B. Madrid J. Miller R. L. Perera F. P.. (2013). Sex-specific epigenetic disruption and behavioral changes following low-dose in utero bisphenol A exposure. Proc. Natl. Acad. Sci. U. S. A. 110, 9956–9961. 10.1073/pnas.1214056110, PMID: 23716699 PMC3683772

[ref31] Kundakovic M. Gudsnuk K. Herbstman J. B. Tang D. Perera F. P. Champagne F. A. (2015). DNA methylation of BDNF as a biomarker of early-life adversity. Proc. Natl. Acad. Sci. U. S. A. 112, 6807–6813. 10.1073/pnas.1408355111, PMID: 25385582 PMC4460453

[ref32] Lee S. W. Chatterjee N. Im J. E. Yoon D. Kim S. Choi J. (2018). Integrated approach of eco-epigenetics and eco-metabolomics on the stress response of bisphenol-A exposure in the aquatic midge *Chironomus riparius*. Ecotoxicol. Environ. Saf. 163, 111–116. 10.1016/j.ecoenv.2018.06.084, PMID: 30041127

[ref33] Li Z. Lyu C. Ren Y. Wang H. (2020). Role of TET dioxygenases and DNA hydroxymethylation in bisphenols-stimulated proliferation of breast cancer cells. Environ. Health Perspect. 128:27008. 10.1289/EHP5862, PMID: 32105160 PMC7064327

[ref34] Lombo M. Getino-Alvarez L. Depince A. Labbe C. Herraez M. P. (2019). Embryonic exposure to bisphenol A impairs primordial germ cell migration without jeopardizing male breeding capacity. Biomol. Ther. 9:307. 10.3390/biom9080307, PMID: 31349731 PMC6722532

[ref35] Mao Z. Xia W. Chang H. Huo W. Li Y. Xu S. (2015). Paternal BPA exposure in early life alters Igf2 epigenetic status in sperm and induces pancreatic impairment in rat offspring. Toxicol. Lett. 238, 30–38. 10.1016/j.toxlet.2015.08.009, PMID: 26276081

[ref36] Matuszczak E. Komarowska M. D. Debek W. Hermanowicz A. (2019). The impact of bisphenol A on fertility, reproductive system, and development: a review of the literature. Int. J. Endocrinol. 2019, 1–8. 10.1155/2019/4068717, PMID: 31093279 PMC6481157

[ref37] Mileva G. Baker S. L. Konkle A. T. Bielajew C. (2014). Bisphenol-A: epigenetic reprogramming and effects on reproduction and behavior. Int. J. Environ. Res. Public Health 11, 7537–7561. 10.3390/ijerph110707537, PMID: 25054232 PMC4113893

[ref38] Morison I. M. Reeve A. E. (1998). A catalogue of imprinted genes and parent-of-origin effects in humans and animals. Hum. Mol. Genet. 7, 1599–1609. 9735381 10.1093/hmg/7.10.1599

[ref39] Olsvik P. A. Whatmore P. Penglase S. J. Skjaerven K. H. Angles d'Auriac M. Ellingsen S. (2019). Associations between behavioral effects of bisphenol A and DNA methylation in zebrafish embryos. Front. Genet. 10:184. 10.3389/fgene.2019.00184, PMID: 30906313 PMC6418038

[ref40] Portela A. Esteller M. (2010). Epigenetic modifications and human disease. Nat. Biotechnol. 28, 1057–1068. 10.1038/nbt.1685, PMID: 20944598

[ref41] Sabry R. Yamate J. Favetta L. LaMarre J. (2019). Micrornas: potential targets and agents of endocrine disruption in female reproduction. J. Toxicol. Pathol. 32, 213–221. 10.1293/tox.2019-0054, PMID: 31719748 PMC6831493

[ref42] Senyildiz M. Karaman E. F. Bas S. S. Pirincci P. A. Ozden S. (2017). Effects of BPA on global DNA methylation and global histone 3 lysine modifications in SH-SY5Y cells: an epigenetic mechanism linking the regulation of chromatin modifiying genes. Toxicol. In Vitro 44, 313–321. 10.1016/j.tiv.2017.07.028, PMID: 28765096

[ref43] Shankar A. Teppala S. Sabanayagam C. (2012). Bisphenol A and peripheral arterial disease: results from the NHANES. Environ. Health Perspect. 120, 1297–1300. 10.1289/ehp.1104114, PMID: 22645278 PMC3440106

[ref44] Singh S. Li S. S. (2012). Epigenetic effects of environmental chemicals bisphenol A and phthalates. Int. J. Mol. Sci. 13, 10143–10153. 10.3390/ijms130810143, PMID: 22949852 PMC3431850

[ref45] Strakovsky R. S. Schantz S. L. (2018). Impacts of bisphenol A (BPA) and phthalate exposures on epigenetic outcomes in the human placenta. Environ. Epigenet. 4:dvy022. 10.1093/eep/dvy022, PMID: 30210810 PMC6128378

[ref46] Suter L. Babiss L. E. Wheeldon E. B. (2004). Toxicogenomics in predictive toxicology in drug development. Chem. Biol. 11, 161–171. 10.1016/j.chembiol.2004.02.003, PMID: 15123278

[ref47] van Esterik J. C. Vitins A. P. Hodemaekers H. M. Kamstra J. H. Legler J. Pennings J. L.. (2015). Liver DNA methylation analysis in adult female c57BL/6JxFVB mice following perinatal exposure to bisphenol A. Toxicol. Lett. 232, 293–300. 10.1016/j.toxlet.2014.10.021, PMID: 25455458

[ref48] Vandenberg L. N. Chahoud I. Heindel J. J. Padmanabhan V. Paumgartten F. J. Schoenfelder G. (2010). Urinary, circulating, and tissue biomonitoring studies indicate widespread exposure to bisphenol A. Environ. Health Perspect. 118, 1055–1070. 10.1289/ehp.0901716, PMID: 20338858 PMC2920080

[ref49] Wang K. Liu S. Svoboda L. K. Rygiel C. A. Neier K. Jones T. R.. (2020). Tissue- and sex-specific DNA methylation changes in mice perinatally exposed to lead (Pb). Front. Genet. 11:840. 10.3389/fgene.2020.00840, PMID: 32973866 PMC7472839

[ref50] Wang T. Han J. Duan X. Xiong B. Cui X. S. Kim N. H.. (2016). The toxic effects and possible mechanisms of bisphenol A on oocyte maturation of porcine in vitro. Oncotarget 7, 32554–32565. 10.18632/oncotarget.8689, PMID: 27086915 PMC5078033

[ref51] Wang T. Pehrsson E. C. Purushotham D. Li D. Zhuo X. Zhang B.. (2018). The NIEHS TaRGET II consortium and environmental epigenomics. Nat. Biotechnol. 36, 225–227. 10.1038/nbt.4099, PMID: 29509741 PMC5991835

[ref52] Wei J. W. Huang K. Yang C. Kang C. S. (2017). Non-coding RNAs as regulators in epigenetics (review). Oncol. Rep. 37, 3–9. 10.3892/or.2016.5236, PMID: 27841002

[ref53] Weinhold B. (2006). Epigenetics: the science of change. Environ. Health Perspect. 114, A160–A167. 10.1289/ehp.114-a160, PMID: 16507447 PMC1392256

[ref54] Weinhouse C. Anderson O. S. Bergin I. L. Vandenbergh D. J. Gyekis J. P. Dingman M. A.. (2014). Dose-dependent incidence of hepatic tumors in adult mice following perinatal exposure to bisphenol A. Environ. Health Perspect. 122, 485–491. 10.1289/ehp.1307449, PMID: 24487385 PMC4014767

[ref55] Weinhouse C. Bergin I. L. Harris C. Dolinoy D. C. (2015). Stat3 is a candidate epigenetic biomarker of perinatal bisphenol A exposure associated with murine hepatic tumors with implications for human health. Epigenetics 10, 1099–1110. 10.1080/15592294.2015.1107694, PMID: 26542749 PMC4844208

[ref56] Weinhouse C. Sartor M. A. Faulk C. Anderson O. S. Sant K. E. Harris C.. (2016). Epigenome-wide DNA methylation analysis implicates neuronal and inflammatory signaling pathways in adult murine hepatic tumorigenesis following perinatal exposure to bisphenol A. Environ. Mol. Mutagen. 57, 435–446. 10.1002/em.22024, PMID: 27334623 PMC4945497

[ref57] Wong R. L. Wang Q. Trevino L. S. Bosland M. C. Chen J. Medvedovic M.. (2015). Identification of secretaglobin Scgb2a1 as a target for developmental reprogramming by BPA in the rat prostate. Epigenetics 10, 127–134. 10.1080/15592294.2015.1009768, PMID: 25612011 PMC4623267

[ref58] Wu X. Huang Q. Javed R. Zhong J. Gao H. Liang H. (2019). Effect of tobacco smoking on the epigenetic age of human respiratory organs. Clin. Epigenet. 11:183. 10.1186/s13148-019-0777-z, PMID: 31801625 PMC6894291

[ref59] Zhu Y. Li Y. Lou D. Gao Y. Yu J. Kong D.. (2018). Sodium arsenite exposure inhibits histone acetyltransferase p300 for attenuating H3K27ac at enhancers in mouse embryonic fibroblast cells. Toxicol. Appl. Pharmacol. 357, 70–79. 10.1016/j.taap.2018.08.011, PMID: 30130555 PMC6526104

